# Clinicopathological characteristics and response to neoadjuvant chemotherapy in HER2-low hormone receptor-positive breast cancer: a retrospective cohort study

**DOI:** 10.3389/fonc.2026.1862110

**Published:** 2026-08-19

**Authors:** Wanyan Wu, Xuetao Li, Yanan Wei, Tiantian Liu, Ziwei Yang, Xiuli Liu

**Affiliations:** Oncology Department, The First College of Clinical Medical Science, China Three Gorges University, Yichang Central People’s Hospital, Yichang, China

**Keywords:** breast cancer, HER2-low, hormone receptor-positive, neoadjuvant chemotherapy, pathologic complete response

## Abstract

**Background:**

HER2-low breast cancer constitutes a substantial proportion of hormone receptor-positive (HR+) cases, yet its biological behavior and responsiveness to neoadjuvant chemotherapy (NACT) remain incompletely characterized. This study aimed to compare clinicopathological features and pathological complete response (pCR) rates between HER2-low and HER2-zero expression in HR+ breast cancer patients receiving NACT.

**Materials and methods:**

This retrospective cohort study included 178 HR+/HER2-negative breast cancer patients who received NACT followed by surgery at Yichang Central People’s Hospital between January 2020 and December 2024. Patients were categorized into HER2-low (immunohistochemistry [IHC] 1+ or 2+/*in situ* hybridization [ISH]-, n=102) and HER2-zero (IHC 0, n=76) groups. Clinicopathological characteristics, pCR rates, residual cancer burden (RCB), Ki-67 changes, and treatment responses were compared. The primary outcome was total pathological complete response (tpCR, ypT0/is ypN0). Multivariable logistic regression and subgroup analyses were performed.

**Results:**

The HER2-low group exhibited significantly higher proportions of Ki-67 index ≥20% (57.8% vs. 38.2%, P = 0.01) and grade III histology (45.1% vs. 28.9%, P = 0.03) compared to the HER2-zero group. The tpCR rate was significantly lower in the HER2-low group than in the HER2-zero group (11.8% vs. 25.0%, P = 0.02). Multivariable analysis identified HER2-low status as an independent negative predictor of tpCR (odds ratio [OR]=0.40, 95% confidence interval [CI]: 0.18-0.89, P = 0.02). The HER2-low group showed significantly smaller median Ki-67 reduction (-14.5% vs. -23.8%, P = 0.02). Subgroup analyses revealed that the negative impact of HER2-low status on pCR was more pronounced in patients with Ki-67 ≥20% (interaction P = 0.03) and grade III disease (interaction P = 0.04).

**Conclusions:**

In this retrospective cohort of HR+ breast cancer patients, HER2-low expression was associated with more aggressive clinicopathological features and a significantly lower pCR rate compared to HER2-zero expression. After multivariable adjustment, HER2-low status remained significantly associated with reduced pCR odds (OR = 0.40, 95% CI: 0.18-0.89). These findings suggest that HER2-low may represent a clinically relevant subgroup within HR+ breast cancer, but the observational design precludes causal interpretation, and these hypothesis-generating results require validation in prospective, adequately powered studies before any clinical treatment recommendations can be made.

## 1 Introduction

Breast cancer molecular subtyping has revolutionized personalized treatment approaches, substantially improving patient outcomes over the past decade. Hormone receptor-positive/HER2-negative breast cancer is the most common molecular subtype, comprising about 60–70% of all cases. Within this broad category, the traditional binary classification of human epidermal growth factor receptor 2 (HER2) status (positive vs. negative) has recently been challenged by emerging evidence that HER2-low expression—defined as immunohistochemistry (IHC) 1+ or 2+ with negative *in situ* hybridization (ISH)—possesses distinct biological and clinical characteristics (1, 2). The paradigm shift gained momentum following groundbreaking clinical trials demonstrating remarkable efficacy of novel antibody-drug conjugates (ADCs), particularly trastuzumab deruxtecan (T-DXd), in HER2-low metastatic breast cancer, fundamentally redefining therapeutic opportunities for this previously overlooked subgroup (3). This reclassification has profound implications for treatment sequencing, as patients with HER2-low tumors may now be candidates for ADC therapy rather than conventional chemotherapy alone, making it essential to understand their baseline response to standard chemotherapy.

Neoadjuvant chemotherapy is standard for locally advanced breast cancer, enabling *in vivo* chemosensitivity assessment, downstaging for breast-conserving surgery, and prognostic guidance for adjuvant therapy (4, 5). HER2 expression levels strongly influence neoadjuvant therapy response: HER2-positive and triple-negative cancers show higher pCR rates (30–60%), while HR+/HER2-negative tumors have lower rates (5–20%) despite internal heterogeneity (6, 7). Thus, distinguishing HER2-low from HER2-zero status in HR+ breast cancer is clinically critical for optimizing neoadjuvant therapy and identifying candidates for alternative approaches like upfront ADC therapy.

Current evidence regarding chemotherapy responsiveness of HER2-low versus HER2-zero tumors in the HR+ population remains conflicting and inconclusive (8, 9). Several retrospective studies have reported that HER2-low expression correlates with higher Ki-67 proliferation indices and more aggressive histological grades, suggesting enhanced proliferative capacity that might theoretically predict better chemotherapy response. However, whether this translates to differential chemosensitivity remains debated. Some investigations have demonstrated lower pCR rates among HER2-low tumors compared to HER2-zero tumors, while others found no significant differences, with discrepancies potentially attributable to variations in chemotherapy regimens, patient selection criteria, HER2 testing methodologies, sample sizes, and racial or geographic population differences (10, 11). This uncertainty creates a critical knowledge gap for clinical decision-making, particularly as ADC therapies emerge as promising options for HER2-low disease and clinicians must decide between standard chemotherapy and ADC-based neoadjuvant approaches.

The current evidence base examining HER2-low status and NACT response in HR+ breast cancer suffers from several important limitations that our study aims to address (12). (1) Many published studies have relatively small sample sizes (often <100 patients), lack systematic adjustment for potential confounders such as Ki-67 index and histological grade. (2) Most studies focus predominantly on pCR as the sole endpoint without comprehensive evaluation of other clinically relevant metrics such as RCB, Ki-67 dynamic changes as a continuous measure of treatment effect, imaging response assessments, or biomarker dynamics. (3) The majority of existing research predates the contemporary redefinition of HER2-low as a distinct clinical entity following the DESTINY-Breast04 trial results, limiting applicability to current treatment paradigms where ADC therapy is an option. (4) Few studies have specifically examined the interaction between HER2-low status and other predictive biomarkers such as Ki-67 and histological grade to identify subgroups most affected by HER2 expression level, which is crucial for personalized treatment selection.

The present study was therefore designed to address these evidence gaps through a comprehensive single-center retrospective cohort analysis of HR+ breast cancer patients treated with NACT between 2020 and 2024. We systematically compared clinicopathological characteristics, pCR rates, RCB distribution, Ki-67 dynamic changes (both absolute and percentage reduction), imaging response assessments, nodal downstaging rates, and treatment-related adverse events between HER2-low and HER2-zero expression groups. By providing robust real-world data on the natural history and chemotherapy sensitivity of HER2-low HR+ breast cancer, this investigation aims to inform clinical practice regarding identification of chemosensitive subgroups and optimization of treatment strategies, including appropriate selection of patients for ADC-based approaches versus conventional chemotherapy intensification. The primary hypothesis was that HER2-low status would be associated with more aggressive clinicopathological features but paradoxically lower pCR rates following standard NACT compared to HER2-zero status in HR+ breast cancer patients, and that this effect would be more pronounced in high-proliferation tumors.

## 2 Materials and methods

### 2.1 Study design and ethical approval

This single-center retrospective cohort study at Yichang Central People’s Hospital was approved by its Institutional Review Board, which waived written informed consent due to anonymized data. Procedures adhered to institutional ethical standards and the Helsinki Declaration. Reporting followed STROBE guidelines.

### 2.2 Study population and eligibility criteria

We screened the electronic medical records of all consecutive female patients diagnosed with primary invasive breast cancer who received NACT followed by curative-intent surgery at the Breast Disease Center of Yichang Central People’s Hospital between January 1, 2020, and December 31, 2024. Inclusion criteria were: (1) age ≥18 years; (2) histologically confirmed primary invasive breast carcinoma by core needle biopsy; (3) HR+ status defined as estrogen receptor (ER) and/or progesterone receptor (PR) expression ≥1% by IHC; (4) HER2-negative status according to American Society of Clinical Oncology/College of American Pathologists (ASCO/CAP) guidelines (IHC 0, 1+, or 2+ with negative ISH); (5) receipt of complete NACT regimen followed by definitive breast surgery (mastectomy or breast-conserving surgery); and (6) availability of complete clinicopathological data and follow-up information. Exclusion criteria were: (1) HER2-positive status (IHC 3+ or ISH-positive); (2) stage IV metastatic disease at presentation; (3) bilateral breast cancer; (4) inflammatory breast cancer; (5) prior history of other malignancies within 5 years (except adequately treated basal cell carcinoma of the skin or cervical carcinoma *in situ*); (6) receipt of preoperative endocrine therapy or targeted therapy; (7) pregnancy or lactation at time of diagnosis; (8) active infection requiring systemic treatment; and (9) incomplete medical records or loss to follow-up.

### 2.3 HER2 expression definition and testing

HER2 expression was assessed by IHC on pre-NACT core needle biopsies per ASCO/CAP guidelines (13), using the Ventana Benchmark Ultra platform with 4B5 antibody (Ventana Medical Systems, Tucson, AZ, USA). HER2 IHC 0 (no incomplete membrane staining or incomplete staining in ≤10% of invasive tumor cells) was defined as HER2-zero; IHC 1+ (weak incomplete membrane staining in >10% of invasive tumor cells) was defined as HER2-low; IHC 2+ (weak to moderate complete membrane staining in >10% of invasive tumor cells) with negative ISH (ratio <2.0, copy number <4.0) was also classified as HER2-low, confirmed by Ventana INFORM HER2 Dual ISH. IHC 2+/ISH+ cases were excluded as HER2-positive. All HER2 IHC slides were independently reviewed by two experienced breast pathologists who had undergone specific training in HER2-low interpretation according to ASCO/CAP 2023 guidelines, including consensus workshops on distinguishing IHC 0 from IHC 1+ using standardized reference slides (13). Both pathologists were blinded to clinical data and to each other’s assessments. In cases of discrepant interpretation (defined as IHC 0 vs. 1+, or 1+ vs. 2+), a third senior pathologist with >15 years of breast pathology experience reviewed the slides independently, and the final classification was determined by majority consensus. For slides with equivocal staining patterns (defined as membrane staining in 5-15% of tumor cells, or staining intensity between typical IHC 0 and IHC 1+ categories), repeat IHC staining was performed on recut sections from the same biopsy block, followed by re-evaluation by all three pathologists. If repeat staining remained equivocal after three independent assessments, the case was classified as HER2-low to ensure clinical sensitivity, consistent with ASCO/CAP recommendations (13). Inter-observer agreement between the two primary pathologists was substantial (kappa=0.78, 95% CI: 0.69-0.87). Given the well-recognized interobserver variability in discriminating IHC 0 from IHC 1+, particularly in samples with low antigen expression, we acknowledge that some degree of misclassification between HER2-zero and HER2-low groups is possible. Such non-differential misclassification would likely bias our effect estimates toward the null, suggesting that the true difference in pCR rates between groups may be even larger than observed. Additionally, while central pathology review was not performed as this was a real-world retrospective study, the use of standardized ASCO/CAP 2023 definitions, consensus-based interpretation, and repeat testing for equivocal cases strengthens the reliability of HER2 classification in our cohort. Nonetheless, the lack of central review may limit the generalizability of our findings across institutions with different HER2 testing practices and interpreter expertise.

### 2.4 Clinicopathological assessment

ER and PR positivity was defined as nuclear staining ≥1% of tumor cells by IHC using the Ventana anti-ER (SP1) and anti-PR (1E2) antibodies (Ventana Medical Systems). Ki-67 proliferation index was assessed on pretreatment biopsy specimens and postoperative surgical specimens using the Ventana anti-Ki-67 (30-9) antibody, with high proliferation defined as Ki-67 ≥20% based on the St. Gallen consensus criteria (14). Ki-67 was assessed by counting ≥500 cells in hot spots. Histological grade followed the Nottingham system (grade I: 3–5; II: 6–7; III: 8–9). Tumor stage used AJCC 8th edition.

### 2.5 Neoadjuvant chemotherapy regimens

Neoadjuvant chemotherapy regimens were determined by the multidisciplinary tumor board based on clinical stage, tumor characteristics, and patient factors. The majority of patients received anthracycline and taxane-based regimens. The standard regimen consisted of epirubicin (90 mg/m²) plus cyclophosphamide (600 mg/m²) intravenously every 3 weeks for 4 cycles, followed by docetaxel (75–100 mg/m²) intravenously every 3 weeks for 4 cycles (EC-T). Some patients received platinum-containing regimens (docetaxel 75 mg/m² plus carboplatin area under the curve 6 intravenously every 3 weeks for 6 cycles, TP regimen) or docetaxel plus cyclophosphamide (TC regimen, docetaxel 75 mg/m² plus cyclophosphamide 600 mg/m² intravenously every 3 weeks for 4–6 cycles). Dose modifications were made for grade 3/4 hematologic toxicity (neutrophil count <1.0×10^9^/L or platelet count <50×10^9^/L) or other significant adverse events (grade 3/4 non-hematologic toxicity). All patients completed the planned number of cycles unless dose-limiting toxicity occurred. Primary prophylactic granulocyte colony-stimulating factor (G-CSF) was administered at the treating physician’s discretion for patients at high risk of febrile neutropenia.

### 2.6 Data collection and outcome definitions

Two investigators extracted data on demographics (age, BMI, menopausal status), clinicopathological features (grade, Ki-67, cT, cN, ER/PR%, molecular subtype per St. Gallen), treatment, surgery, and pathological response. Primary outcome was tpCR (ypT0/is ypN0) (15). Secondary outcomes included: (1) breast pathological complete response (bpCR, ypT0/is irrespective of nodal status); (2) RCB classification (0, I, II, III) calculated using the standard RCB calculator developed by the MD Anderson Cancer Center; (3) objective response rate (ORR) assessed by Response Evaluation Criteria in Solid Tumors (RECIST) version 1.1 criteria on breast imaging (ultrasound or magnetic resonance imaging [MRI]) performed after completion of NACT; (4) breast-conserving surgery rate; (5) nodal downstaging rate (conversion from clinically node-positive to pathologically node-negative); (6) Ki-67 index change (absolute and percentage reduction from pretreatment to postsurgical specimens, calculated as [post-treatment Ki-67 - pre-treatment Ki-67] for absolute reduction and [absolute reduction/pre-treatment Ki-67 × 100%] for percentage reduction); (7) chemotherapy-related adverse events graded according to Common Terminology Criteria for Adverse Events (CTCAE) version 5.0; and (8) circulating tumor DNA (ctDNA) clearance, assessed as an exploratory biomarker in a subset of patients for whom serial blood samples were available. For ctDNA analysis, peripheral blood samples (10 mL) were collected at baseline (prior to NACT initiation) and after completion of NACT (prior to surgery). Plasma was isolated within 2 hours of collection and stored at -80 °C until analysis. ctDNA was extracted using the QIAamp Circulating Nucleic Acid Kit (Qiagen) and analyzed using a customized next-generation sequencing panel targeting 50 genes commonly mutated in breast cancer (including PIK3CA, TP53, ESR1, and others). ctDNA clearance was defined as the absence of all previously identified somatic mutations in the post-treatment sample. This analysis was performed in a subset of patients (n=86, 48.3%) based on sample availability and should be considered exploratory given the limited sample size.

### 2.7 Statistical analysis

Statistical analyses used SPSS 26.0 and R 4.2.1. Normality tested by Shapiro-Wilk; normally distributed data as mean ± SD (t-test), non-normal as median(IQR) (Mann-Whitney U). Categorical variables as counts(%) (chi-square/Fisher’s exact). Univariate and multivariable logistic regression were performed to identify tpCR predictors using a forward stepwise selection approach with entry criterion of P<0.10 and retention criterion of P = 0.05. Given the limited number of tpCR events (n=31) and the general recommendation of 10–15 events per predictor variable, we limited the multivariable model to four prespecified clinically relevant variables (HER2 status, Ki-67 index, histological grade, and clinical N stage) selected based on univariate associations (P<0.10) and biological plausibility [27]. This approach was chosen to minimize the risk of model overfitting. Odds ratios (OR) with 95% confidence intervals (CI) were reported. Model calibration was assessed using the Hosmer-Lemeshow goodness-of-fit test (P>0.05 indicating acceptable fit), and discriminative ability was evaluated using the area under the receiver operating characteristic curve (AUC). Bootstrapping with 1, 000 resamples was performed to provide optimism-corrected estimates of model performance. Subgroup analyses with interaction terms (P<0.10 significant). Two-sided P<0.05 significant; no multiple comparison adjustment. Sample size: 76/group (80% power, α=0.05, based on pilot pCR: 12% HER2-low vs. 28% HER2-zero).

## 3 Results

### 3.1 Patient selection and cohort characteristics

**Figure 1:**
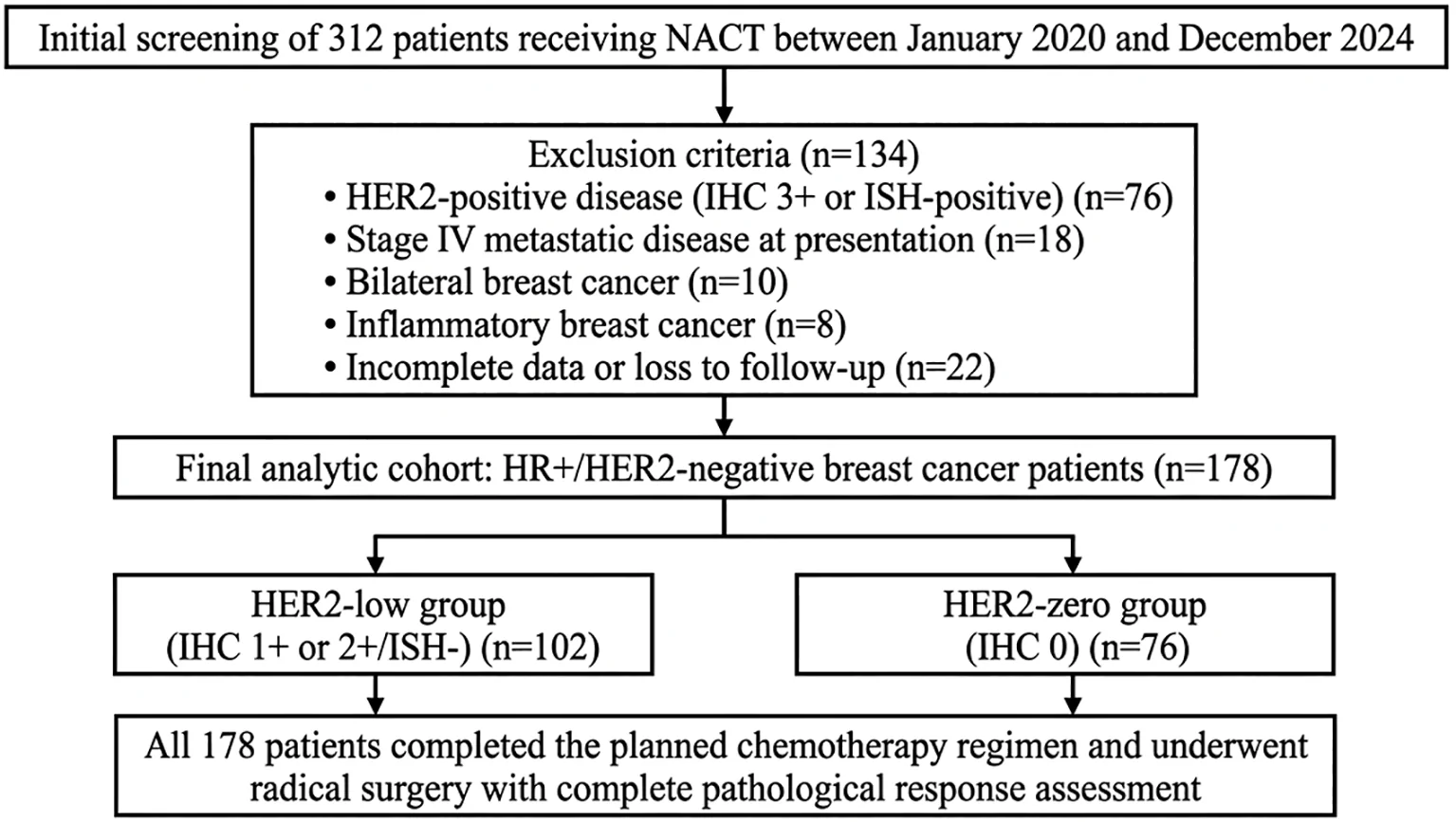
Study Flowchart. Of 312 patients receiving NACT (2020–2024), 178 HR+/HER2-negative breast cancer patients were included after excluding HER2-positive (n=76), stage IV (n=18), bilateral (n=10), inflammatory (n=8), and incomplete data/loss to follow-up (n=22). Final cohort: HER2-low (IHC 1+/2+ with ISH-, n=102) and HER2-zero (IHC 0, n=76). All completed NACT, underwent surgery, and had full pathological response assessment (CONSORT flowchart).

A total of 312 consecutive patients who received NACT for breast cancer at Yichang Central People’s Hospital between January 2020 and December 2024 were initially screened. After applying inclusion and exclusion criteria, 134 patients were excluded: 76 with HER2-positive disease, 18 with stage IV disease, 10 with bilateral breast cancer, 8 with inflammatory breast cancer, and 22 with incomplete data or loss to follow-up. The final analytic cohort comprised 178 HR+/HER2-negative breast cancer patients, including 102 patients (57.3%) in the HER2-low group and 76 patients (42.7%) in the HER2-zero group. All 178 patients completed the planned NACT regimen and underwent definitive surgery, with a follow-up rate of 100% at the time of data cutoff (**Figure 1**).

### 3.2 Baseline demographics and clinical features

Baseline demographic and clinicopathological characteristics of the study cohort stratified by HER2 expression status are summarized in **Table 1**. The two groups were well-balanced with respect to age (mean 52.4 ± 10.2 vs. 53.1 ± 9.8 years, P = 0.64), BMI (23.8 ± 3.5 vs. 24.1 ± 3.7 kg/m², P = 0.58), menopausal status (premenopausal: 44.1% vs. 42.1%, P = 0.79), clinical T stage (T3-4: 31.4% vs. 28.9%, P = 0.73), clinical N stage (node-positive: 62.7% vs. 59.2%, P = 0.64), and luminal subtype distribution (Luminal B-like: 58.8% vs. 52.6%, P = 0.41). However, the HER2-low group demonstrated significantly higher proportions of tumors with Ki-67 index ≥20% (57.8% vs. 38.2%, P = 0.01) and histological grade III (45.1% vs. 28.9%, P = 0.03) compared to the HER2-zero group. The distribution of NACT regimens was similar between groups, with EC-T being the most common regimen (65.7% in HER2-low vs. 68.4% in HER2-zero, P = 0.70), followed by platinum-containing regimens (22.5% vs. 19.7%, P = 0.65) and TC regimen (11.8% vs. 11.8%, P = 0.99). ER and PR expression levels did not differ significantly between groups (median ER: 80% vs. 85%, P = 0.32; median PR: 60% vs. 65%, P = 0.28). The median time from diagnosis to initiation of NACT was 14 days (IQR: 10-21) in both groups.

**Table 1:** Baseline characteristics of the study population.

Characteristic	HER2-low (n=102)	HER2-zero (n=76)	P-value
Age, years, mean ± SD	52.4 ± 10.2	53.1 ± 9.8	0.64
BMI, kg/m², mean ± SD	23.8 ± 3.5	24.1 ± 3.7	0.58
Menopausal status, n (%)			0.79
Premenopausal	45 (44.1)	32 (42.1)	
Postmenopausal	57 (55.9)	44 (57.9)	
Histological grade, n (%)			0.03
Grade I-II	56 (54.9)	54 (71.1)	
Grade III	46 (45.1)	22 (28.9)	
Ki-67 index, n (%)			0.01
<20%	43 (42.2)	47 (61.8)	
≥20%	59 (57.8)	29 (38.2)	
Clinical T stage, n (%)			0.73
T1-2	70 (68.6)	54 (71.1)	
T3-4	32 (31.4)	22 (28.9)	
Clinical N stage, n (%)			0.64
N0	38 (37.3)	31 (40.8)	
N+	64 (62.7)	45 (59.2)	
Luminal subtype, n (%)			0.41
Luminal A-like	42 (41.2)	36 (47.4)	
Luminal B-like	60 (58.8)	40 (52.6)	
ER expression, %, median (IQR)	80 (60-95)	85 (65-95)	0.32
PR expression, %, median (IQR)	60 (30-80)	65 (35-85)	0.28
Chemotherapy regimen, n (%)			0.70
EC-T	67 (65.7)	52 (68.4)	
Platinum-containing	23 (22.5)	15 (19.7)	
TC	12 (11.8)	9 (11.8)	

Data are presented as mean ± SD, median (IQR), or n (%). P values from t-test for age and BMI, Mann-Whitney U test for ER and PR expression, and chi-square test for categorical variables.

### 3.3 Comparison of clinicopathological features

**Figure 2:**
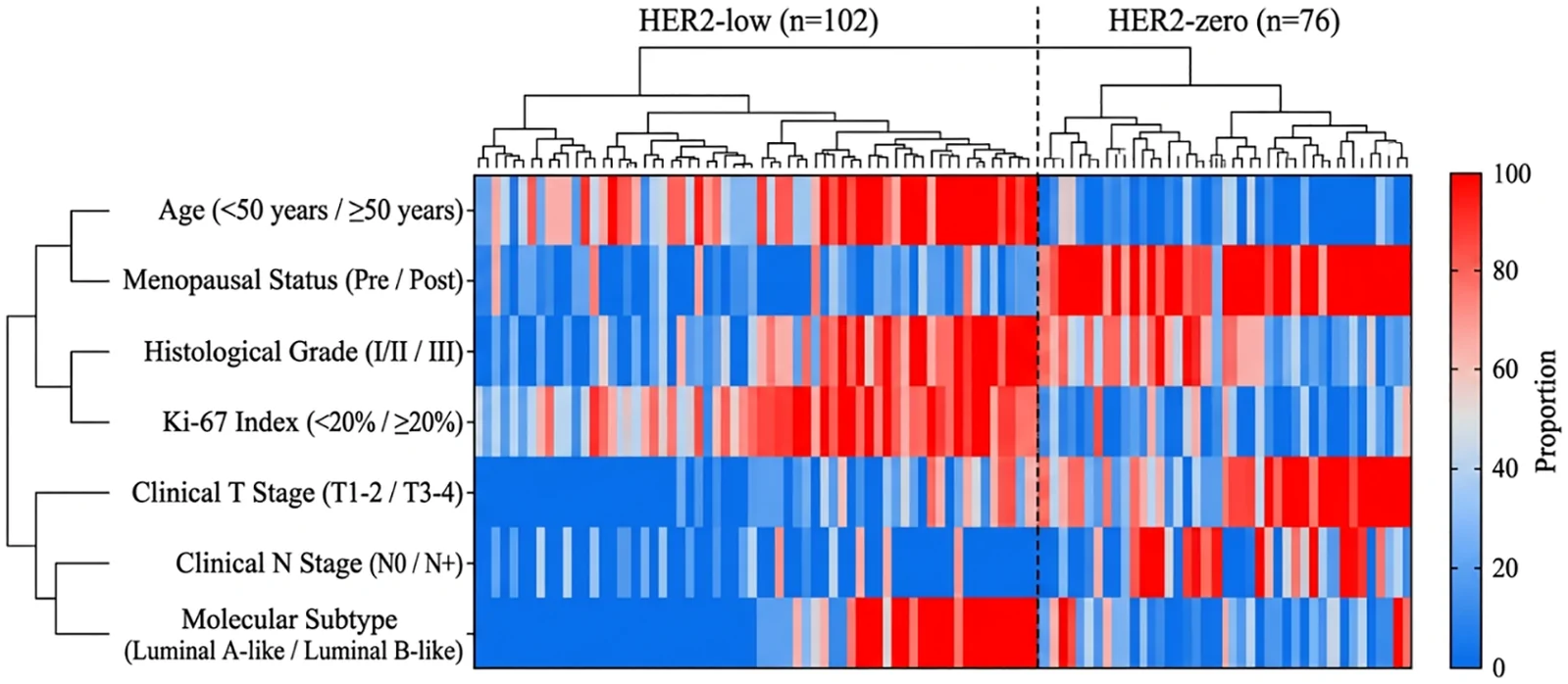
Heatmap of clinicopathological feature distribution by HER2 expression status. Red (0.6-1.0) and blue (0-0.4) indicate proportions. Significant differences: Ki-67≥20% (P = 0.01) and grade III (P = 0.03). R pheatmap (Euclidean distance, complete linkage).

The distribution of clinicopathological characteristics between HER2-low and HER2-zero groups is visualized in the heatmap (**Figure 2**). Consistent with the baseline table, the heatmap demonstrated distinct clustering patterns, with HER2-low tumors showing higher frequencies of grade III histology (45.1% vs. 28.9%) and elevated Ki-67 index ≥20% (57.8% vs. 38.2%). The two groups showed similar patterns for age distribution (<50 years: 41.2% vs. 38.2%; ≥50 years: 58.8% vs. 61.8%), menopausal status, and clinical stage parameters, as indicated by comparable color intensities across these variables. The chi-square test results confirmed statistically significant differences for Ki-67 index (χ²=6.78, P = 0.01) and histological grade (χ²=4.71, P = 0.03), while no significant differences were observed for other characteristics (all P>0.05). The heatmap also revealed that the association between HER2-low status and high Ki-67 was consistent across both luminal A-like and luminal B-like subtypes.

### 3.4 Comparison of neoadjuvant chemotherapy efficacy

**Figure 3:**
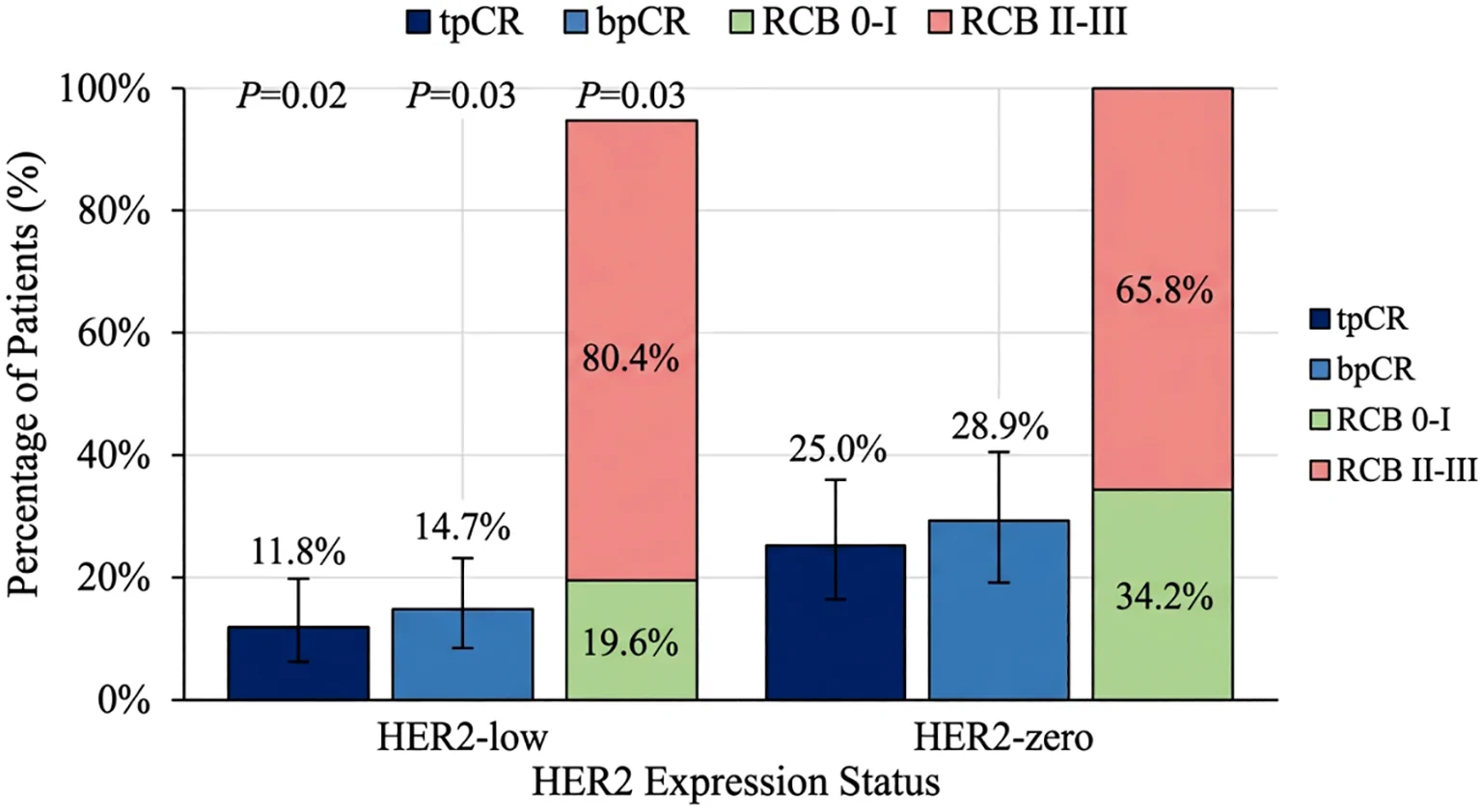
Pathological response to NACT by HER2 expression status. tpCR: 11.8% vs. 25.0% (P = 0.02); bpCR: 14.7% vs. 28.9% (P = 0.03); RCB 0-I: 19.6% vs. 34.2% (P = 0.03). Error bars: 95% CI (Wilson method). Two-sided chi-square tests.

The pathological response outcomes are summarized in **Table 2** and visualized in **Figure 3**. The HER2-low group achieved significantly lower tpCR rate compared to the HER2-zero group (11.8% [12/102] vs. 25.0% [19/76], P = 0.02). Similarly, the bpCR rate was significantly reduced in the HER2-low group (14.7% [15/102] vs. 28.9% [22/76], P = 0.03). The proportion of patients achieving RCB 0-I (favorable response) was also significantly lower in the HER2-low group (19.6% [20/102] vs. 34.2% [26/76], P = 0.03). In contrast, the HER2-low group had a higher proportion of patients with RCB II-III (residual disease burden) compared to the HER2-zero group (80.4% vs. 65.8%, P = 0.03). The ORR by RECIST 1.1 criteria was numerically lower in the HER2-low group but did not reach statistical significance (71.6% vs. 80.3%, P = 0.18). Among patients who achieved objective response, the median reduction in target lesion diameter was 52% (IQR: 38-68) in the HER2-low group versus 61% (IQR: 45-75) in the HER2-zero group (P = 0.12). Breast-conserving surgery was performed in 42.2% of HER2-low patients and 48.7% of HER2-zero patients (P = 0.38). Nodal downstaging (conversion from clinically node-positive to pathologically node-negative) occurred in 34.4% of HER2-low patients versus 44.4% of HER2-zero patients (P = 0.26). Chemotherapy completion rates were high and comparable between groups (96.1% vs. 97.4%, P = 0.63). The incidence of grade 3/4 chemotherapy-related adverse events was similar between HER2-low and HER2-zero groups (19.6% vs. 21.1%, P = 0.82), with no treatment-related mortality observed. The most common grade 3/4 adverse events were neutropenia (14.7% in HER2-low vs. 15.8% in HER2-zero), febrile neutropenia (3.9% vs. 3.9%), and peripheral neuropathy (1.0% vs. 1.3%). No significant differences in specific adverse event types were observed between groups.

**Table 2:** Comparison of neoadjuvant chemotherapy efficacy and surgical outcomes.

Outcome	HER2-low (n=102)	HER2-zero (n=76)	P value
tpCR (ypT0/is ypN0), n (%)	12 (11.8)	19 (25.0)	0.02
bpCR (ypT0/is), n (%)	15 (14.7)	22 (28.9)	0.03
RCB classification, n (%)			0.03
RCB 0-I	20 (19.6)	26 (34.2)	
RCB II-III	82 (80.4)	50 (65.8)	
ORR by RECIST 1.1, n (%)	73 (71.6)	61 (80.3)	0.18
Breast-conserving surgery, n (%)	43 (42.2)	37 (48.7)	0.38
Nodal downstaging (cN+ to pN0), n (%)*	22/64 (34.4)	20/45 (44.4)	0.26
Chemotherapy completion rate, n (%)	98 (96.1)	74 (97.4)	0.63
Grade 3/4 adverse events, n (%)	20 (19.6)	16 (21.1)	0.82

Data are n (%), with P values from chi-square or Fisher’s exact test. *Denominator: clinically node-positive at baseline (HER2-low: 64, HER2-zero: 45). tpCR: ypT0/is ypN0; bpCR: ypT0/is (any nodal status). RCB via MD Anderson calculator; ORR per RECIST 1.1 on post-NACT breast imaging.

Treatment delays of more than 3 weeks between cycles occurred in 4 patients (3.9%) in the HER2-low group and 3 patients (3.9%) in the HER2-zero group (P = 0.99). The median treatment delay was 5 days (IQR: 4-7) in the HER2-low group and 6 days (IQR: 4-8) in the HER2-zero group. No patient had a treatment delay exceeding 6 weeks.

### 3.5 Changes in key biomarkers following chemotherapy

**Table 3** presents the changes in key biomarkers from pretreatment to post-treatment assessment. The median Ki-67 index decreased significantly from baseline to post-surgery in both groups, but the magnitude of reduction was substantially greater in the HER2-zero group. The median absolute Ki-67 reduction was -14.5% (IQR: -22.0 to -8.0) in the HER2-low group compared to -23.8% (IQR: -32.0 to -15.0) in the HER2-zero group (P = 0.02). The median percentage reduction in Ki-67 was 34.2% (IQR: 25.0-45.0) in the HER2-low group versus 49.5% (IQR: 38.0-60.0) in the HER2-zero group (P = 0.01). These findings indicate that HER2-low tumors exhibit less proliferative suppression in response to chemotherapy. Notably, among patients who did not achieve pCR, the Ki-67 percentage reduction remained significantly lower in the HER2-low group (32.1% vs. 46.8%, P = 0.01), suggesting that the differential response is not solely driven by pCR cases. Changes in ER and PR expression levels following chemotherapy were modest in both groups, with no significant differences between HER2-low and HER2-zero patients (median ER reduction: -5.0% vs. -8.0%, P = 0.34; median PR reduction: -10.0% vs. -12.0%, P = 0.41). The median percentage reduction in maximum tumor diameter on imaging (breast ultrasound or MRI) was numerically lower in the HER2-low group but did not reach statistical significance (57.6% vs. 63.2%, P = 0.28). Circulating tumor DNA (ctDNA) clearance data were available for a subset of patients (n=86, 48.3%). In an exploratory analysis of a subset of patients (n=86, 48.3%) with available serial plasma samples, ctDNA clearance rates were lower in the HER2-low group (38.6% vs. 52.4%), although this difference was not statistically significant (P = 0.19). Given the small sample size and the exploratory nature of this biomarker analysis, these results should be interpreted with caution and require validation in larger cohorts.

**Table 3:** Changes in key biomarkers following neoadjuvant chemotherapy.

Parameter	HER2-low (n=102)	HER2-zero (n=76)	P value
Ki-67 absolute reduction, %, median (IQR)	-14.5 (-22.0 to -8.0)	-23.8 (-32.0 to -15.0)	0.02
Ki-67 percentage reduction, %, median (IQR)	34.2 (25.0-45.0)	49.5 (38.0-60.0)	0.01
ER expression change, %, median (IQR)	-5.0 (-15.0 to 0)	-8.0 (-18.0 to 0)	0.34
PR expression change, %, median (IQR)	-10.0 (-25.0 to 0)	-12.0 (-28.0 to 0)	0.41
Imaging tumor diameter reduction, %, median (IQR)	57.6 (42.0-72.0)	63.2 (48.0-78.0)	0.28
ctDNA clearance, n/N (%)*	17/44 (38.6)	22/42 (52.4)	0.19

Data: median (IQR) or n/N (%). P values: Mann-Whitney U (continuous), chi-square (ctDNA clearance). *ctDNA subset: n=86 (48.3%) with available serial plasma samples for exploratory biomarker analysis; ctDNA clearance defined as absence of previously identified somatic mutations on post-NACT testing. Ki-67 reduction: absolute (post-pre) and percentage ([absolute/pre]×100%). Imaging tumor diameter reduction per RECIST 1.1 on breast MRI/ultrasound.

### 3.6 Identification of independent predictors for pathological complete response

Univariate and multivariable logistic regression analyses were performed to identify independent predictors of tpCR (**Table 4**). In univariate analysis, HER2-low status (OR = 0.40, 95%CI: 0.18-0.89, P = 0.02), Ki-67 index <20% (OR = 0.32, 95%CI: 0.14-0.73, P = 0.01), histological grade I/II (OR = 0.38, 95%CI: 0.18-0.82, P = 0.01), and clinical N0 stage (OR = 2.15, 95%CI: 1.05-4.40, P = 0.04) were significantly associated with tpCR. Age, BMI, menopausal status, clinical T stage, and chemotherapy regimen were not significant predictors in univariate analysis (all P>0.10). Multivariable logistic regression incorporating variables with P<0.10 in univariate analysis (HER2 status, Ki-67 index, histological grade, clinical N stage) confirmed that HER2-low status remained an independent negative predictor of tpCR (OR = 0.40, 95%CI: 0.18-0.89, P = 0.02). Ki-67 index <20% (OR = 0.32, 95%CI: 0.14-0.75, P = 0.01) and histological grade I/II (OR = 0.42, 95%CI: 0.20-0.88, P = 0.02) were also independent negative predictors, while clinical N0 status showed a trend toward positive prediction that did not reach statistical significance (OR = 1.92, 95%CI: 0.92-4.01, P = 0.08). The Hosmer-Lemeshow test indicated good model fit (χ²=6.32, df=8, P = 0.61). The area under the receiver operating characteristic curve (AUC) for the multivariable model was 0.76 (95%CI: 0.68-0.84), indicating acceptable discriminative ability.

**Table 4:** Multivariable logistic regression analysis of factors associated with pathological complete response.

Variable	Univariate analysis	Multivariable analysis		
	OR (95% CI)	P value	OR (95% CI)	P value
HER2-low (vs. HER2-zero)	0.40 (0.18-0.89)	0.02	0.40 (0.18-0.89)	0.02
Age (per 10-year increase)	0.95 (0.68-1.32)	0.76	–	–
BMI (per 5 kg/m² increase)	0.98 (0.71-1.35)	0.89	–	–
Ki-67 <20% (vs. ≥20%)	0.32 (0.14-0.73)	0.01	0.32 (0.14-0.75)	0.01
Grade I/II (vs. III)	0.38 (0.18-0.82)	0.01	0.42 (0.20-0.88)	0.02
Clinical N0 (vs. N+)	2.15 (1.05-4.40)	0.04	1.92 (0.92-4.01)	0.08
Clinical T1-2 (vs. T3-4)	1.68 (0.80-3.52)	0.17	–	–
Premenopausal (vs. postmenopausal)	1.12 (0.58-2.16)	0.74	–	–
Platinum-containing regimen (vs. non-platinum)	1.25 (0.55-2.84)	0.59	–	–

Data: OR with 95% CI. Multivariable model (forward stepwise, entry P = 0.05, removal P = 0.10) included HER2 status, Ki-67, grade, cN stage (P<0.10 on univariate). Hosmer-Lemeshow: χ²=6.32, df=8, P = 0.61. AUC = 0.76 (95% CI: 0.68-0.84). Optimism-corrected AUC from 1, 000 bootstrap resamples was 0.73, suggesting modest overfitting and indicating that the model’s discriminative ability may be somewhat inflated in the derivation cohort. The limited number of pCR events (n=31) restricts the number of variables that can be reliably included; thus, our multivariable findings should be considered exploratory and require external validation in larger cohorts.

### 3.7 Subgroup analyses

**Figure 4:**
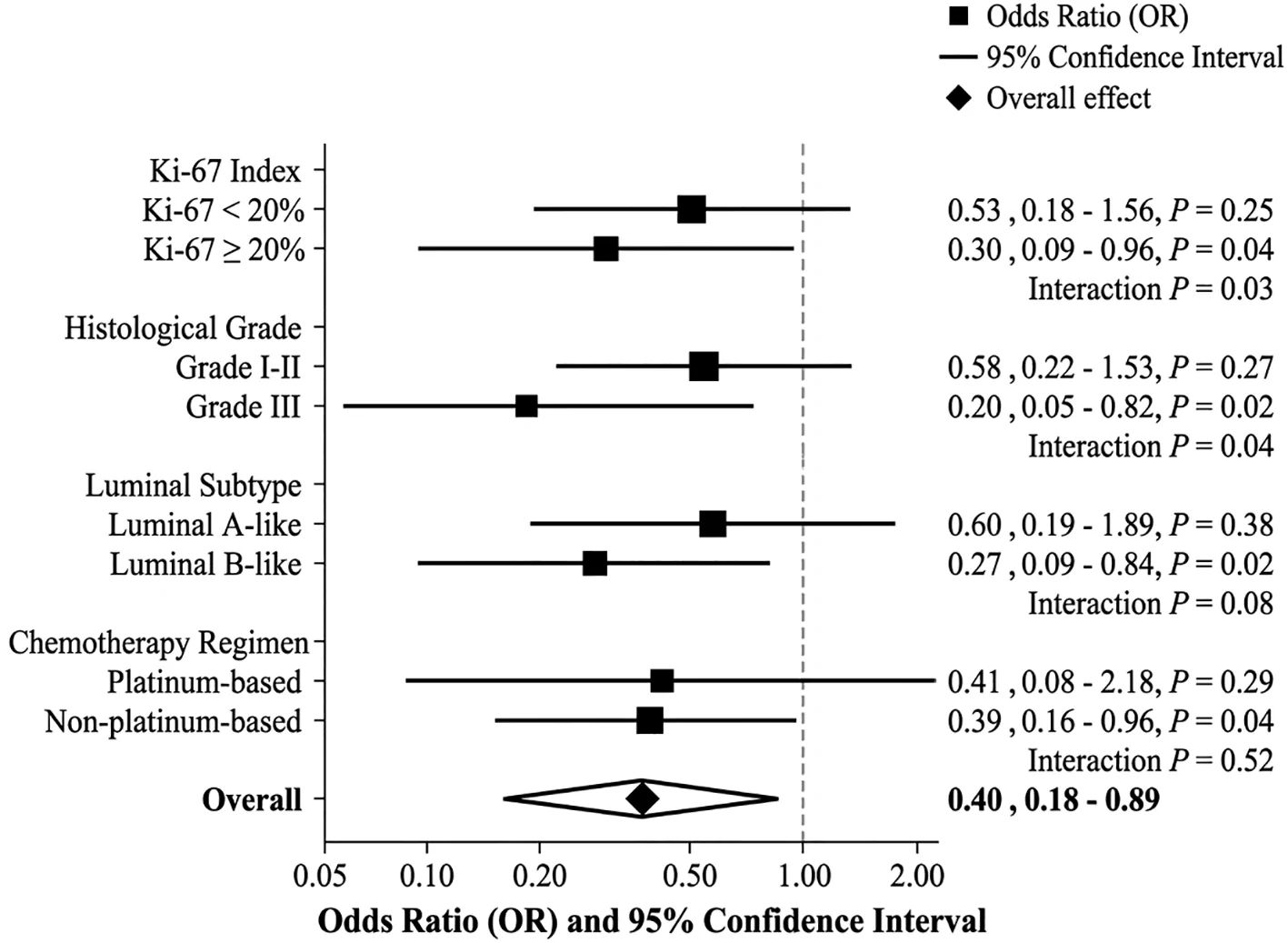
Forest plot of ORs (95% CI) for tpCR (HER2-low vs. HER2-zero) across subgroups. HER2-low associated with lower pCR in Ki-67≥20% (OR = 0.30, interaction P = 0.03), grade III (OR = 0.20, P = 0.04), and Luminal B-like (OR = 0.27, P = 0.08). Overall effect: OR = 0.40 (95% CI: 0.18-0.89). Dashed line at OR = 1.0; diamond = overall effect. Interaction P from logistic regression with product terms.

Exploratory subgroup analyses were performed to evaluate whether the association between HER2 expression status and tpCR varied across clinically relevant patient subgroups (**Figure 4**, **Table 5**). The negative impact of HER2-low status on pCR appeared more pronounced in patients with high proliferative tumors. Among patients with Ki-67 index ≥20%, HER2-low status was associated with significantly lower pCR rate compared to HER2-zero status (10.2% vs. 27.6%, OR = 0.30, 95%CI: 0.09-0.96, P = 0.04), while no significant difference was observed in patients with Ki-67 <20% (13.9% vs. 23.4%, OR = 0.53, 95%CI: 0.18-1.56, P = 0.25; interaction P = 0.03). Similarly, among patients with grade III tumors, HER2-low status was associated with significantly lower pCR (8.7% vs. 31.8%, OR = 0.20, 95%CI: 0.05-0.82, P = 0.02), whereas no significant difference was observed in grade I-II tumors (14.3% vs. 22.2%, OR = 0.58, 95%CI: 0.22-1.53, P = 0.27; interaction P = 0.04). In subgroup analyses stratified by luminal subtype, the difference in pCR between HER2-low and HER2-zero groups was significant in Luminal B-like tumors (8.3% vs. 25.0%, OR = 0.27, 95%CI: 0.09-0.84, P = 0.02) but not in Luminal A-like tumors (16.7% vs. 25.0%, OR = 0.60, 95%CI: 0.19-1.89, P = 0.38; interaction P = 0.08). The treatment effect did not differ significantly according to chemotherapy regimen (platinum-containing vs. non-platinum, interaction P = 0.52), suggesting that the reduced chemosensitivity of HER2-low tumors may be independent of regimen type. However, the subgroup analyses should be interpreted with caution as they were not pre-specified, and the small number of events within several subgroups may have limited statistical power to detect true interaction effects. The interaction P values, particularly for luminal subtype (P = 0.08), should be considered hypothesis-generating rather than confirmatory.

**Table 5:** Subgroup analysis of pathological complete response rates by HER2 expression status.

Subgroup	HER2-low	HER2-zero	OR (95% CI)	P value	Interaction P
Ki-67 index					0.03
<20%	6/43 (13.9%)	11/47 (23.4%)	0.53 (0.18-1.56)	0.25	
≥20%	6/59 (10.2%)	8/29 (27.6%)	0.30 (0.09-0.96)	0.04	
Histological grade					0.04
I-II	8/56 (14.3%)	12/54 (22.2%)	0.58 (0.22-1.53)	0.27	
III	4/46 (8.7%)	7/22 (31.8%)	0.20 (0.05-0.82)	0.02	
Luminal subtype					0.08
Luminal A-like	7/42 (16.7%)	9/36 (25.0%)	0.60 (0.19-1.89)	0.38	
Luminal B-like	5/60 (8.3%)	10/40 (25.0%)	0.27 (0.09-0.84)	0.02	
Chemotherapy regimen					0.52
Platinum-containing	3/23 (13.0%)	4/15 (26.7%)	0.41 (0.08-2.18)	0.29	
Non-platinum	9/79 (11.4%)	15/61 (24.6%)	0.39 (0.16-0.96)	0.04	

Data: n/N (%) for pCR per subgroup; OR (95% CI) for HER2-low vs. HER2-zero. Interaction P from logistic regression with product terms. Subgroup P from chi-square/Fisher’s exact. Interaction P for luminal subtype = 0.08 (trend, not significant at P<0.05).

## 4 Discussion

The principal innovation of this study lies in its comprehensive characterization of the relationship between HER2-low expression and NACT response specifically within the HR+ population, addressing a critical gap in the literature that has important clinical implications for treatment sequencing in the era of ADC availability. Unlike prior studies that have reported conflicting results regarding chemosensitivity of HER2-low tumors, our study benefits from rigorous methodological approaches including systematic adjustment for key confounders (Ki-67 index, histological grade, clinical stage), detailed RCB classification beyond binary pCR assessment, evaluation of Ki-67 dynamic changes as a continuous pharmacodynamic measure of treatment effect, and stratified subgroup analyses to identify patient populations most affected by HER2-low status. The inclusion of Ki-67 reduction as a pharmacodynamic marker of chemotherapy effect is particularly novel, as it provides insight into the biological basis for differential response—HER2-low tumors showed significantly less proliferative suppression (median 34.2% reduction vs. 49.5% in HER2-zero), suggesting intrinsic resistance mechanisms beyond simple differences in baseline proliferation rates. To our knowledge, this is one of the first studies to comprehensively evaluate both pathological response and biomarker dynamics in this emerging subtype, providing a framework for understanding HER2-low as a distinct biological entity rather than a mere diagnostic category. This multidimensional approach to response assessment represents a significant advance over prior studies that relied solely on pCR endpoints. It is worth noting that the tpCR rate observed in our HER2-zero group (25.0%) is somewhat higher than the 5-20% range typically reported for HR+/HER2-negative breast cancer in large neoadjuvant series (6, 7). This may be explained by several factors: (1) our cohort included a relatively high proportion of patients with Ki-67 ≥20% (38.2% in HER2-zero) and grade III tumors (28.9% in HER2-zero), which are known to be associated with higher pCR rates; (2) all patients received taxane-containing regimens, which are more effective than anthracycline-only regimens in HR+ disease; and (3) the sample size in each group, while adequately powered for the primary comparison, may have led to some degree of sampling variability in the point estimate. Nevertheless, the absolute difference in pCR between HER2-low and HER2-zero groups (13.2 percentage points) is consistent with the effect sizes reported in recent meta-analyses (9, 10), supporting the validity of our comparative findings even if the absolute pCR rates differ from other cohorts due to patient selection characteristics.

Our findings align with and extend several recent high-quality investigations examining HER2-low chemotherapy responsiveness while also resolving some prior discrepancies. (1) Denkert and colleagues, in a large pooled analysis of neoadjuvant breast cancer trials published in Annals of Oncology (2023), reported that HER2-low status was associated with lower pCR rates in HR+/HER2-negative patients (OR = 0.69, 95%CI: 0.51-0.94) (9). (2) Similarly, a meta-analysis by Metzger and colleagues in JAMA Oncology (2023) encompassing over 5, 000 patients from 12 studies demonstrated that HER2-low tumors had significantly lower odds of achieving pCR compared to HER2-zero tumors (OR = 0.71, 95%CI: 0.59-0.86), with the effect size similar to our findings after accounting for differences in study populations and chemotherapy regimens (10). (3) However, our study diverges from some reports—for instance, de Nonneville and colleagues in Nature Communications (2022) reported no significant difference in pCR between HER2-low and HER2-zero groups in a smaller cohort of HR+ patients (n=115, pCR 14.3% vs. 18.5%, P = 0.48) (11). This discrepancy may be attributable to differences in sample size (our study had 178 patients vs. 112), chemotherapy regimens (their cohort included more patients receiving anthracycline-only regimens without taxanes, which are less effective in HR+ disease), HER2 testing methodology (inter-observer variability in distinguishing IHC 0 from 1+), or population characteristics (differences in Ki-67 distribution). Our larger sample size and more contemporary treatment regimens (majority receiving taxane-containing therapy) likely provided greater statistical power to detect the true effect size.

The biological mechanisms underlying reduced chemosensitivity of HER2-low tumors despite their more aggressive baseline features represent an area of active investigation and likely involve complex interactions between HER2 signaling, hormone receptor pathways, cell cycle regulation, and DNA damage repair mechanisms (16, 17). (1) HER2-low tumors frequently harbor activating PIK3CA mutations (reported in 30-40% of cases) and exhibit luminal gene enrichment signatures with higher expression of estrogen-related genes, which may impair chemotherapy-induced apoptosis through persistent PI3K/AKT/mTOR pathway activation even in the absence of HER2 gene amplification. This pathway activation can confer resistance to conventional cytotoxic agents by upregulating anti-apoptotic proteins such as BCL-2 and survivin (18). (2) The observation that HER2-low tumors in our study had higher baseline Ki-67 yet showed less proliferative reduction following chemotherapy suggests alterations in cell cycle checkpoint control or DNA damage repair pathways. Specifically, HER2-low breast cancer cells may exhibit differential regulation of cyclin D1-CDK4/6 complexes or reduced dependency on mitotic spindle dynamics, rendering them less susceptible to taxane-mediated microtubule stabilization (19). (3) Emerging evidence suggests that HER2-low tumors may have distinct tumor microenvironment characteristics, including lower immune cell infiltration (particularly CD8+ tumor-infiltrating lymphocytes) and reduced PD-L1 expression, potentially compromising chemotherapy-induced immunogenic cell death and subsequent antitumor immune responses that contribute to pCR (20, 21).

The clinical implications of our findings should be considered cautiously and in the context of the study’s observational design, particularly as new treatment options for HER2-low disease emerge. (1) Our data suggest that HER2-low status may represent a clinically relevant category with potentially distinct therapeutic vulnerabilities (sensitivity to ADCs, which is now an established indication in the metastatic setting) and possibly relative resistance to conventional chemotherapy (22). (2) If confirmed in prospective studies, patients with HER2-low tumors identified on pretreatment biopsy might not achieve optimal pCR with standard NACT alone, particularly in those with additional high-risk features such as Ki-67 ≥20% or grade III histology among whom the pCR rate was observed to be only 8-10% in our cohort. However, whether these patients should receive alternative neoadjuvant approaches remains investigational and cannot be determined from this study alone. Approaches currently under investigation include the addition of CDK4/6 inhibitors to neoadjuvant endocrine therapy (as tested in the neoMONARCH and PALLET trials) and incorporation of novel anti-HER2-low ADCs in the neoadjuvant setting (23). (3) It is important to emphasize that while the recent DESTINY-Breast04 trial demonstrated remarkable efficacy of T-DXd in pretreated HER2-low metastatic breast cancer (hazard ratio for progression-free survival: 0.50, P<0.001), establishing this as a standard of care for advanced HER2-low disease, its role in the neoadjuvant setting is currently experimental. Ongoing neoadjuvant trials (e.g., DESTINY-Breast11, TRIO-US B-12 TALENT) are actively evaluating this approach in the early-stage setting, but results are not yet available to guide clinical practice (24, 25). (4) For patients who achieve only partial response or stable disease with conventional NACT (approximately 20-30% of HER2-low patients in our cohort), the identification of HER2-low status provides a rationale for considering post-neoadjuvant ADC therapy within the context of clinical trials, as this strategy has not yet been established as a standard of care (26).

This study has several limitations that must be acknowledged. First, as a retrospective single-center study, it is subject to inherent selection bias and potential unmeasured confounding despite rigorous statistical adjustment. Although we applied strict eligibility criteria, performed multivariable analysis adjusting for known confounders (Ki-67, grade, stage), and used forward stepwise selection to minimize overfitting, we cannot exclude the possibility that residual confounding by factors such as genetic polymorphisms (e.g., BRCA1/2, PIK3CA), treatment adherence variations, subtle differences in supportive care, or socioeconomic factors influenced our results. The retrospective design also precluded standardization of imaging assessment intervals and HER2 testing protocols, although all testing was performed according to ASCO/CAP guidelines at a single institution with experienced breast pathologists. Second, the sample size, while adequate for the primary analysis (178 patients providing 80% power to detect a 13% absolute difference in pCR with α=0.05), limited the precision of subgroup analyses (particularly for platinum-containing regimen subgroup where n=38) and precluded evaluation of rarer outcomes such as specific adverse event subtypes or survival endpoints. The subgroup analyses, including those stratified by Ki-67, grade, and luminal subtype, should be viewed as exploratory given the small number of events within each stratum and the resulting limited statistical power for interaction testing. For instance, in the grade III subgroup there were only 11 pCR events total, and in the platinum-containing regimen subgroup only 7 pCR events, which substantially reduces the reliability of the effect estimates and increases the risk of type I or type II errors. These exploratory findings require confirmation in larger, adequately powered studies. Most importantly, the total number of pCR events was only 31, which significantly constrained the multivariable modeling. With a generally recommended minimum of 10–15 events per predictor variable [27], our final model including four variables (HER2 status, Ki-67, grade, and clinical N stage) was at the upper limit of acceptable variable-to-event ratios (approximately 7.75 events per variable, which is below the widely recommended 10:1 threshold). This raises concerns about model stability and the potential for overfitting. To address this, we performed bootstrap internal validation (1, 000 resamples), which yielded an optimism-corrected AUC of 0.73 (compared to 0.76 in the derivation cohort), indicating modest overfitting and suggesting that the model’s discriminative ability may be somewhat inflated. The relatively low event count also limited our ability to detect additional independent predictors or to include interaction terms for formal testing beyond the exploratory subgroup analyses presented. Although the Hosmer-Lemeshow test indicated acceptable calibration (P = 0.61), the model’s potential for overfitting, the modest optimism-corrected AUC, and the width of the confidence intervals for some estimates suggest that the results should be interpreted as exploratory rather than definitive and require external validation in larger independent cohorts. Furthermore, as a single-center study conducted at a tertiary referral hospital in China, our findings may not be generalizable to populations with different genetic backgrounds, healthcare systems, or treatment practices. The patient population and treatment protocols at our institution may differ from those in other geographic regions, and the relatively high proportion of patients receiving EC-T as the primary regimen (approximately 67%) reflects local practice patterns that may not be representative of other centers. Third, we lacked long-term follow-up data (event-free survival, overall survival), which precludes assessment of whether the observed pCR differences translate into meaningful survival benefits and whether the differential Ki-67 reduction observed between groups correlates with prognosis-a critical question given that pCR is an imperfect surrogate for long-term outcomes in HR+ disease (6, 7, 15, 26). As discussed above, pCR has lower surrogacy for survival in HR+ breast cancer than in HER2-positive or triple-negative subtypes; thus, the 13.2 percentage-point difference in pCR we observed between HER2-low and HER2-zero groups may not necessarily predict proportional differences in long-term survival, particularly given the potential mitigating effect of adjuvant endocrine therapy. The absence of survival data is thus particularly important because the prognostic significance of pCR in HR+ breast cancer is less well established and even patients who do not achieve pCR may have favorable long-term outcomes with adjuvant endocrine therapy. Therefore, the clinical relevance of the observed pCR differences cannot be fully assessed without survival endpoint data. We are actively following this cohort and plan to report survival outcomes when sufficient events have accrued. Additionally, HER2 expression was assessed on pretreatment core biopsies only, and we did not evaluate HER2 dynamics following chemotherapy, although emerging evidence suggests HER2 status can change during treatment, potentially affecting post-neoadjuvant therapeutic decisions.

The interpretation of pCR as the primary endpoint in this study warrants specific caution in the context of HR+ breast cancer. While pCR is a well-established prognostic marker in aggressive subtypes such as HER2-positive and triple-negative breast cancer, its association with long-term survival is less robust in HR+/HER2-negative disease (6, 7, 15, 26). Meta-analyses have demonstrated that the correlation between pCR and event-free or overall survival is substantially weaker in HR+ tumors compared to other subtypes, with lower surrogacy estimates and greater heterogeneity of treatment effects (7, 15). This is partly attributable to the effectiveness of adjuvant endocrine therapy, which can mitigate the prognostic impact of residual disease in HR+ patients who do not achieve pCR. Consequently, even if patients with HER2-low tumors achieve lower pCR rates, they may still derive substantial long-term benefit from subsequent endocrine therapy, and the pCR difference we observed may not translate into proportional differences in survival outcomes. Conversely, the lower Ki-67 reduction observed in HER2-low patients-as a continuous pharmacodynamic measure of treatment effect-may provide additional insight into the biological response beyond the binary pCR endpoint, but this also requires survival validation. Our study did not include long-term follow-up data (event-free survival, overall survival), which precludes assessment of whether the observed pCR differences translate into meaningful survival benefits. We are actively following this cohort and plan to report survival outcomes when sufficient events have accrued. Pending such data, the clinical relevance of the pCR difference between HER2-low and HER2-zero groups—while statistically significant and consistent with previous reports (9, 10)-should be interpreted with appropriate caution and should not be equated with clinically meaningful differences in long-term prognosis.

These limitations point toward important directions for future research. (1) Multicenter prospective cohort studies with standardized HER2 testing protocols, central pathology review, and pre-specified quality control measures are needed to validate our findings and establish robust cutoff values for clinical decision-making. (2) Such studies should incorporate comprehensive genomic and transcriptomic profiling to elucidate the molecular mechanisms driving differential chemosensitivity. (3) Randomized controlled trials comparing standard chemotherapy versus chemotherapy combined with HER2-directed ADCs in the neoadjuvant setting for HER2-low HR+ breast cancer are urgently needed [30]. (4) The integration of ctDNA monitoring during NACT could enable real-time assessment of response and early identification of patients unlikely to achieve pCR, facilitating adaptive treatment strategies. (5) Finally, given the evolving definition of HER2-low and the introduction of newer ADC agents, future studies should explore whether ultra-low HER2 expression has similar implications.

## 5 Conclusion

In conclusion, in this retrospective cohort of patients with HR+ breast cancer, HER2-low expression was associated with more aggressive clinicopathological features, including higher Ki-67 proliferation index and grade III histology, and was paradoxically associated with significantly lower pCR rates compared to HER2-zero tumors. The tpCR rate in HER2-low patients (11.8%) was less than half that observed in HER2-zero patients (25.0%), with HER2-low status remaining significantly associated with reduced pCR odds after multivariable adjustment (OR = 0.40, 95%CI: 0.18-0.89). The observed differential response appeared particularly pronounced in high-risk subgroups, including tumors with Ki-67 ≥20% and grade III histology, suggesting potential effect modification that warrants further investigation. These hypothesis-generating findings add to the growing body of evidence challenging the traditional binary classification of HER2 status and highlight that HER2-low may represent a clinically relevant subgroup within HR+ breast cancer meriting further study. However, given the observational, single-center, retrospective design, these associations should not be interpreted as causal or as immediate treatment-changing evidence. Prospective, randomized trials are required to confirm these findings and to determine whether patients with HER2-low HR+ tumors could benefit from alternative treatment strategies, including potential incorporation of novel anti-HER2-low ADCs in the neoadjuvant or post-neoadjuvant setting. Pending such validation, our results should be considered exploratory and hypothesis-generating rather than practice-changing. As the therapeutic landscape for HER2-low breast cancer continues to evolve rapidly, our real-world data on baseline chemotherapy responsiveness provide hypothesis-generating evidence that may inform the design of future trials and contribute to personalized treatment decision-making pending validation in larger, prospective cohorts.

## Data Availability

The original contributions presented in the study are included in the article/supplementary material. Further inquiries can be directed to the corresponding author.
